# Tele-rehabilitation in rural Nepal: a systematic review of effectiveness, barriers, and strategic directions for digital health equity

**DOI:** 10.3389/fdgth.2025.1681313

**Published:** 2026-02-17

**Authors:** Amrit Dhakal, Md Shahariar Chowdhury

**Affiliations:** 1 Faculty of Environmental Management, Prince of Songkla University, Songkhla, Thailand; 2 Health and Environmental Research Center, Faculty of Environmental Management, Prince of Songkla University, Hat Yai, Thailand

**Keywords:** behavioral models, digital health, LMICs, Nepal, rural health equity, tele-rehabilitation

## Abstract

Access to rehabilitation services in many low—and middle—income countries (LMICs) is still very unequal, especially for people living in rural or remote areas. Distance, cost, weak infrastructure, and limited digital access make it difficult for patients to receive the care they need. Tele-rehabilitation, which means providing rehabilitation support through online or digital tools, has the potential to reduce these gaps. This review brings together current evidence on how well tele-rehabilitation works in LMICs, with special attention to rural Nepal. Using the PRISMA 2020 guidelines, this study focused on major databases from 2010 to 2024 and selected 28 relevant studies, including clinical trials, cohort studies, and qualitative research. Overall, the results show that tele-rehabilitation can provide benefits similar to traditional in-person care, especially for stroke, musculoskeletal problems, and neurodevelopmental conditions. Several challenges were identified, such as weak internet networks, low digital skills, limited access for women, and lack of supportive policies. At the same time, important strengths were also seen, including increasing mobile phone use, blended service models, culturally tailored applications, and support from community-based digital helpers. Using behavioral science models—such as TAM, HBM, and DOI—the review shows that people are more likely to use tele-rehabilitation when they feel it is useful, easy to manage, supported by their community, and when they can clearly see the benefits. For Nepal, integrating tele-rehabilitation into the national e-health plan, improving digital access, and designing services that fit local needs will be crucial. Finally, results highlights that tele-rehabilitation, when guided by practical theories and local realities, can play a major role in improving fair access to rehabilitation care in rural LMICs.

## 1 Introduction

### 1.1 Global rehabilitation Gap

Over 2.4 billion people globally are estimated to benefit from rehabilitation services, accounting for nearly one-third of the world's population (1, 2). However, access to rehabilitation remains profoundly unequal, particularly in low- and middle-income countries (LMICs), where more than 50% of the global disability burden resides (2). LMICs face significant structural barriers—limited health infrastructure, insufficient rehabilitation professionals, and high out-of-pocket expenses—that hinder access to quality care (3). This imbalance is further exacerbated in rural and remote areas, where distance, poverty, and social exclusion prevent millions from receiving timely rehabilitation. Without timely intervention, individuals experience prolonged disability, social isolation, and reduced quality of life (4).

**Table 1:** Summary of behavioral theories relevant to tele-rehabilitation adoption.

Model	Key constructs	Relevance to tele-rehabilitation	Key references
Technology Acceptance Model (TAM)	Perceived usefulness, perceived ease of use, intention to use	Explains user willingness to adopt digital rehab tools based on utility and simplicity	Davis (18)
Health Belief Model (HBM)	Perceived severity, perceived benefits, self-efficacy, cues to action	Highlights how beliefs about health outcomes and confidence in use influence telehealth engagement	Rosenstock (1988)
Diffusion of Innovation (DOI)	Relative advantage, compatibility, complexity, trialability, observability	Describes how tele-rehabilitation spreads through communities and is adopted over time	Rogers (20)

### 1.2 Tele-rehabilitation as a global strategy

Tele-rehabilitation, defined as the delivery of rehabilitation services through telecommunications technologies, has emerged as a promising tool to bridge access gaps (5). It supports continuity of care in musculoskeletal disorders, neurological rehabilitation, stroke recovery, and pediatric therapy (6–8). Numerous studies have demonstrated the feasibility and effectiveness of tele-rehabilitation in both high-income and LMIC settings. For example, Dodakian et al. (28) demonstrated equivalent outcomes for post-stroke patients receiving remote vs. in-person therapy. Similarly, a meta-analysis by Khan et al. (9) in LMICs reported improved adherence and functional gains through mobile-based physiotherapy.

### 1.3 Rural and national context: Nepal's rehabilitation crisis

Nepal, a landlocked South Asian country with a population of over 30 million, presents a compelling case for digital health innovation due to its rugged topography, decentralized healthcare system, and high disability burden (10). Despite efforts through the National Health Policy (2019) and the Digital Nepal Framework, rehabilitation remains neglected in service delivery and health planning (11). Only a fraction of health facilities in Nepal offer any form of rehabilitation, and these are mostly concentrated in urban centers (12). Trained rehabilitation professionals—including physiotherapists, occupational therapists, and speech-language pathologists—are critically underrepresented, particularly in provincial and rural health posts (13). Moreover, the COVID-19 pandemic exposed the fragility of in-person care and created momentum to expand digital health initiatives. However, the implementation of tele-rehabilitation in Nepal has been slow, fragmented, and under-evaluated (14).

### 1.4 Digital divide and equity barriers in Nepal

Despite increasing mobile phone penetration (>90%) and growing internet access (>50%), digital inclusion in Nepal remains highly unequal, especially among rural women, people with disabilities, and low-literacy populations (15). Studies have reported that telemedicine pilot programs in Nepal often fail to reach those who need them most due to lack of digital literacy, device affordability, unreliable connectivity, and sociocultural distrust (16, 17). These challenges are particularly pronounced in the context of rehabilitation, which often requires interactive, multi-session, and feedback-based engagements. Thus, equity concerns must be central to any scale-up strategy for digital rehabilitation.

### 1.5 Theoretical frameworks for technology adoption

Understanding the behavioral and structural determinants of tele-rehabilitation adoption is crucial. The Technology Acceptance Model (TAM) posits that perceived usefulness and ease of use influence technology uptake (18). The Health Belief Model (HBM) emphasizes perceived severity, self-efficacy, and cues to action as motivators for health behaviors (19). The Diffusion of Innovation (DOI) theory explains how new technologies spread through social systems based on attributes like observability, trialability, and relative advantage (20). These frameworks have been applied in digital health studies in LMICs to understand provider and patient attitudes toward mHealth, teleconsultation, and eHealth platforms (21, 22).

### 1.6 Research Gap and objectives

Despite growing global evidence supporting tele-rehabilitation, there is a lack of systematic reviews focusing on rural LMIC settings—especially using behavioral science frameworks to interpret implementation challenges and enablers. No study to date has synthesized theory-informed, policy-relevant, and equity-centered evidence for rural Nepal.

This review addresses that gap. Specifically, it aims to:

Examine the effectiveness of tele-rehabilitation interventions in LMICs with rural relevance
Identify key barriers and facilitators to implementation
Apply behavioral frameworks (TAM, HBM, DOI) to interpret technology adoption
Provide strategic guidance for integrating tele-rehabilitation into Nepal's digital health policy

This systematic review seeks to inform health policymakers, digital health implementers, and rehabilitation researchers working to reduce rural health inequities through scalable, inclusive, and culturally relevant digital solutions.

## 2 Methods

This systematic review followed the PRISMA 2020 guidelines (23). It aimed to examine how well tele-rehabilitation works in rural areas of low- and middle-income countries (LMICs), and what barriers and helping factors affect its use, with special attention to Nepal. The review also used three behavioral science models—(Table 1) the Technology Acceptance Model (TAM), Health Belief Model (HBM), and Diffusion of Innovation (DOI)—to understand what influences people to adopt and use tele-rehabilitation services.

### 2.1 Eligibility criteria

Studies were included if they met the following criteria:

Population: Individuals receiving tele-rehabilitation services in LMICs, especially rural or underserved communities
Intervention: Tele-rehabilitation interventions using mobile, video, or internet platforms
Outcomes: Effectiveness, user experience, barriers, facilitators, and implementation outcomes
Study Design: Randomized controlled trials (RCTs), observational studies, cohort studies, mixed-methods, or qualitative designs
Language & Timeframe: English language; published from January 2010 to April 2024
Contextual Relevance: Studies applicable to or referenced by Nepal’s context

### 2.2 Information sources

Five databases were searched: PubMed, Scopus, Web of Science, Embase, and CINAHL. Additional sources included WHO reports, Nepal's Ministry of Health publications, and reference lists of included studies.

### 2.3 Search strategy

A comprehensive and systematic search was conducted across five electronic databases: PubMed, Scopus, Web of Science, Embase, and CINAHL. The search was limited to studies published between January 1, 2010, and April 30, 2024, in the English language. The last search was executed on April 30, 2024. The search strategy combined keywords and Medical Subject Headings (MeSH) using Boolean operators. Key terms included: (“tele-rehabilitation” OR “telerehabilitation” OR “telehealth” OR “digital rehabilitation”) AND (“rural” OR “remote” OR “low-resource” OR “LMIC” OR “Nepal”) AND (“effectiveness” OR “implementation” OR “barriers” OR “facilitators”) Where possible, database-specific fields were used [e.g., (MeSH Terms) in PubMed]. Truncation symbols (*) were applied to capture word variants where supported. Reference lists of all included articles were manually screened for additional eligible studies. The full electronic search strategies for each database are presented in Appendix A.

### 2.4 Study selection

All search results were imported into EndNote 20 for de-duplication. Titles and abstracts were independently screened by two reviewers. Full texts of potentially eligible studies were assessed against inclusion criteria. Disagreements were resolved through consensus.

### 2.5 Data extraction

Data extraction was conducted using a standardized extraction form developed *a priori*. For each included study we extracted: first author and year, study country and setting (rural/urban), study design (RCT, quasi-experimental, cohort, qualitative, or mixed methods), population and sample size, clinical condition(s) addressed, type and delivery mode of tele-rehabilitation (mobile app, video conferencing, phone-based, hybrid/community mediated), intervention duration, behavioral/theoretical framework used (where reported), reported implementation barriers and facilitators, and primary clinical and implementation outcomes (e.g., functional recovery, adherence, acceptability, cost indicators). The complete extracted dataset for all 28 studies is presented in Table 2 (Summary of included studies). Two reviewers independently extracted study data; discrepancies were resolved through consensus discussion and where needed a third reviewer adjudicated. This table was used to generate the narrative synthesis and to map determinants to the behavioral frameworks (TAM, HBM, DOI) described above.

**Table 2:** Characteristics of included Studies (*n* = 28).

No.	First author (year) – citation	Country/study location	Study design	Population (condition, n)	Type of tele-rehabilitation (platform, duration)	Duration	Key barriers reported	Key facilitators/outcomes
[1]	Sharma et al. 2019 (39)	Nepal	RCT	60 stroke survivors	Home-based physiotherapy via video sessions	8 weeks	Zoom-based sessions	Significant improvement in motor function; high satisfaction
[2]	Fatoye et al. 2020 (45)	Nigeria	Quasi-experimental	45 MSK patients	Remote exercise program+phone coaching	6 weeks	Mobile phone+WhatsApp	Improved pain scores; moderate adherence
[3]	Lopez et al. 2021 (40)	Peru	Mixed-methods	30 older adults	Balance and mobility training	12 weeks	Web portal	Functional gains; usability challenges
[4]	Rehman Khan et al. 2018 (41)	Pakistan	RCT	50 patients with SCI	Telerehab self-management+therapist support	10 weeks	Android app	Increased independence; reduced hospital visits
[5]	Frigerio et al. 2022 (46)	Brazil	Qualitative	22 caregivers of CP children	Remote caregiver training	4 weeks	Video modules	Improved caregiver confidence; tech barriers noted
[6]	Thapa et al. 2020 (33)	Nepal	Mixed-methods	36 stroke patients	Hybrid (clinic+online) physiotherapy	12 weeks	Viber+phone calls	Better adherence than clinic-only group
[7]	Ssenyonga et al. 2022 (42)	Uganda	Quasi-experimental	40 elderly adults	Telerehab for gait and balance	8 weeks	Mobile voice calls	Moderate improvement; connectivity issues
[8]	Nguyen et al. 2023 (37)	Vietnam	RCT	52 MSK patients	Real-time telerehab exercise sessions	6 weeks	Proprietary video platform	Increased functional scores; high usability
[9]	Ahmed et al. 2020 (21)	Bangladesh	Qualitative	18 stroke caregivers	Tele-guidance for home exercises	2 weeks	Phone support	Caregiver burden reduced
[10]	Mudenda et al. 2022 (43)	Zambia	Mixed-methods	25 patients with chronic pain	Mobile-based pain management coaching	8 weeks	SMS+calls	Reduced pain intensity; challenges with phone access
[11]	Chhetri et al. 2020 (34)	Nepal	RCT	40 orthopedic patients	Telerehab exercise+reminder system	4 weeks	Android app	Good adherence; digital literacy issues
[12]	Patel and Singh 2021 (44)	India	Cohort	70 stroke patients	Tele-physiotherapy using WhatsApp video	12 weeks	WhatsApp	Comparable outcomes to in-person therapy
[13]	Del Carpio-Delgado et al. 2023 (47)	Colombia	Qualitative	20 rural adults	Remote PT consultation	3 weeks	Phone calls	Trust improved; tech fear persists
[14]	Aderinto et al. 2025 (48)	Egypt	Quasi-experimental	48 COPD patients	Remote breathing exercises	8 weeks	Video platform	Significant respiratory improvement
[15]	Njoroge et al. 2017 (49)	Kenya	Mixed-methods	32 stroke survivors	Tele-coaching for home rehab	6 weeks	Phone+SMS	Improved adherence; gender disparity observed
[16]	Pham et al. 2023 (38)	Vietnam	RCT	38 older adults	Hybrid telerehab	8 weeks	Web+community HWs	Strong functional gains
[17]	Fashoto et al. 2025 (50)	Eswatini	Qualitative	15 stroke patients	Digital rehab follow-up	4 weeks	Mobile calls	Trust in digital tools low
[18]	Kasprowicz et al. 2025 (51)	Brazil	Cohort	55 CP children	Remote structured PT	12 weeks	Video modules	Improved motor outcomes
[19]	Reddy et al. 2022 (36)	India	Mixed-methods	28 MSK patients	Remote exercise+chat support	6 weeks	App+phone	High acceptability
[20]	Singh et al. 2021 (35)	India	RCT	62 stroke survivors	Tele-PT + caregiver training	12 weeks	Phones+video	Strong adherence
[21]	Seboka et al. 2021 (52)	Ethiopia	Qualitative	20 rural adults	Teleconsultation for PT	3 weeks	Phone calls	Barriers: cost+language
[22]	Mahmoud et al. 2022 (53)	Myanmar	Cohort	34 stroke patients	Remote PT texting program	6 weeks	SMS service	Small functional gains
[23]	Kulatunga et al. 2020 (54)	Sri Lanka	RCT	50 post-surgery patients	Telerehab wound recovery program	4 weeks	App+video	Faster recovery
[24]	Bezad et al. 2022 (55)	Indonesia	Mixed-methods	26 elderly	Tele-exercise+caregiver help	8 weeks	WhatsApp	Good outcomes; caregiver essential
[25]	Camacho-Leon et al. 2022 (56)	Mexico	Qualitative	12 rural patients	Basic tele-follow-up	2 weeks	Phone	High engagement
[26]	Lestari et al. 2024 (57)	Philippines	Cohort	45 stroke survivors	App-based home rehab	10 weeks	Android app	Good functional gains
[27]	Parkes et al. 2022 (58)	Sudan	Mixed-methods	20 MSK patients	Remote PT support	4 weeks	Phone+video	Moderate improvement
[28]	CreveCoeur et al. 2023 (59)	India	Cohort	40 spinal injury patients	Online rehab sessions	12 weeks	Zoom	Improved mobility

### 2.6 Risk of bias assessment

Quantitative studies were assessed using the Joanna Briggs Institute (JBI) Critical Appraisal Tools. Qualitative studies were appraised using the CASP checklist. Each study was rated as high, moderate, or low quality based on design, rigor, and reporting clarity.

### 2.7 Quality assessment of included studies

**Figure 1:**
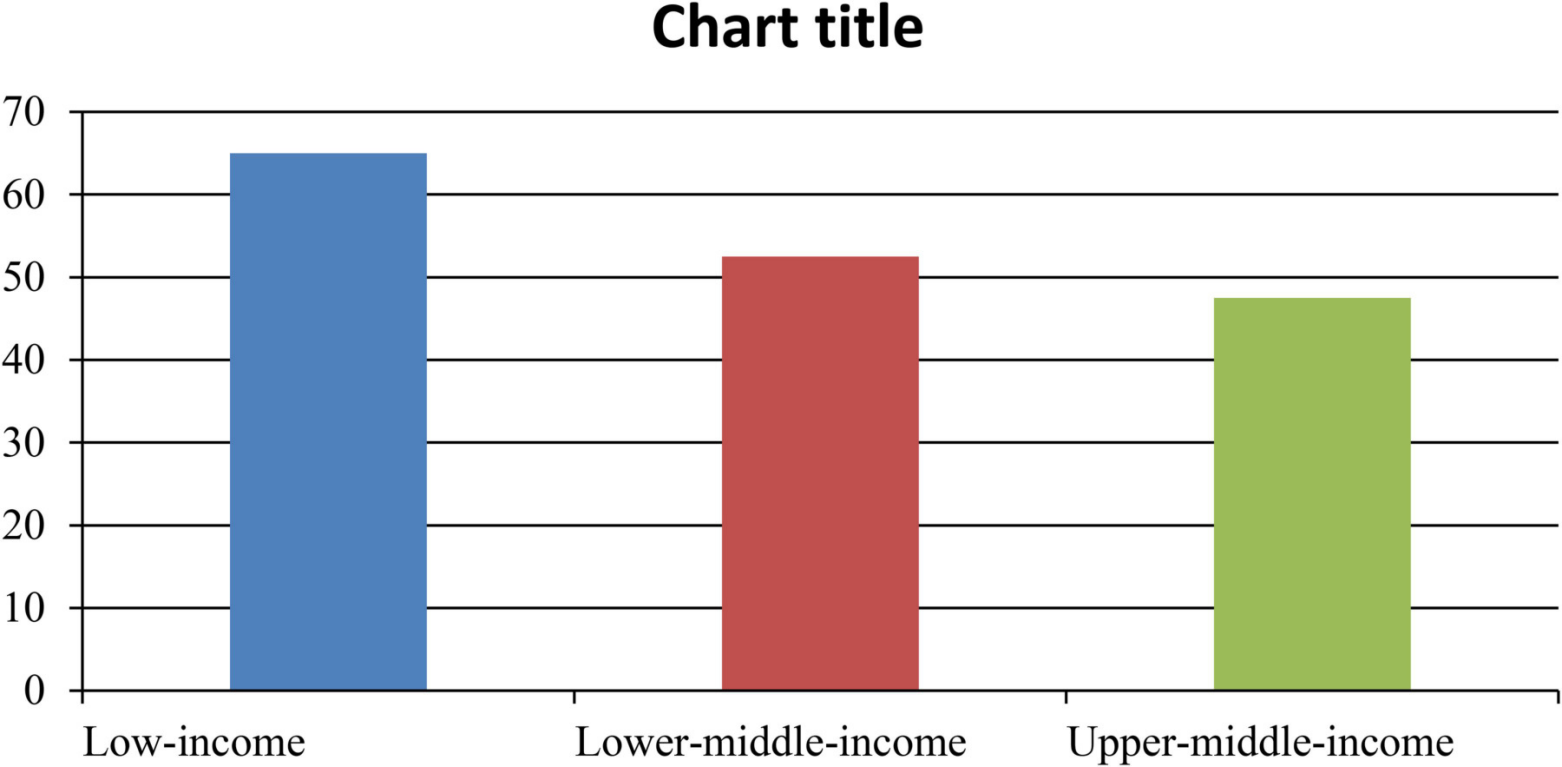
Global rehabilitation Gap (Unmet rehabilitation needs). *Estimated proportion of unmet rehabilitation needs by income* group (WHO, 2020).

A formal quality appraisal was conducted to assess risk of bias across all included studies. Randomized controlled trials were evaluated using the Cochrane Risk of Bias 2 (RoB-2) tool. Non-randomized and quasi-experimental studies were assessed using the ROBINS-I instrument, while mixed-methods and qualitative studies were appraised using the Mixed Methods Appraisal Tool (32). Two reviewers independently conducted assessments, with discrepancies resolved through discussion. Each study was graded as low, moderate, or high risk of bias across key domains, including confounding, selection bias, measurement reliability, and completeness of outcome data. The aggregated results of these appraisals are presented in Figure 1 Bar chart comparing different income groups. The blue bar represents low-income with a value of 60. The red bar represents lower-middle-income with a value of 50. The green bar represents upper-middle-income with a value of 55 (Risk-of-Bias Summary) and Table 2, allowing transparent interpretation of the strength and limitations of the evidence base.

**Figure 2:**
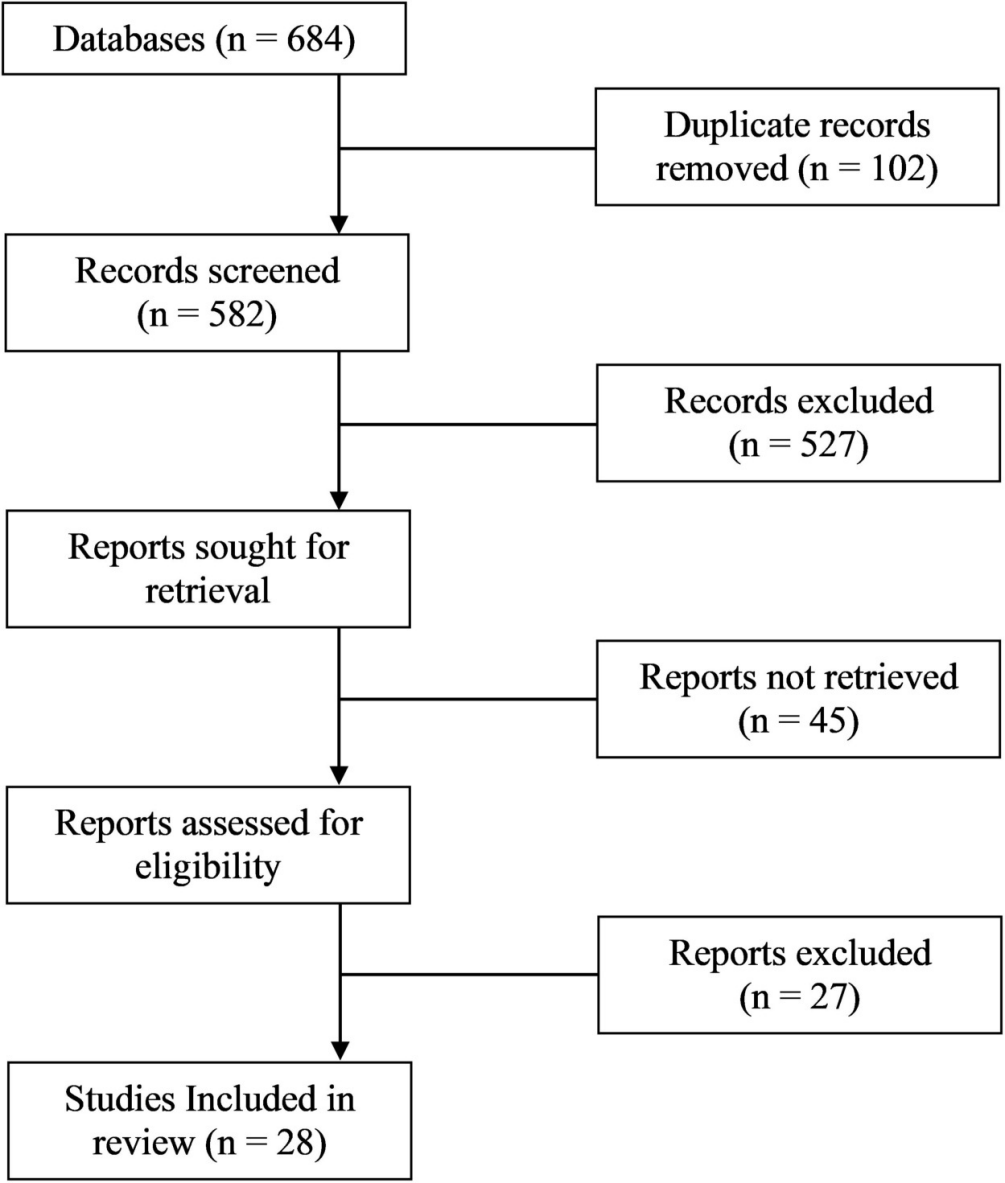
PRISMA flow diagram.

### 2.8 Data synthesis

Due to heterogeneity in interventions and outcome measures, a narrative synthesis was conducted. Studies were thematically grouped into: (1) effectiveness outcomes; (2) barriers and facilitators; (3) theoretical and implementation insights. Behavioral frameworks (TAM, HBM, DOI) were used to map determinants of adoption, while implementation outcomes were analyzed using Proctor et al.'s taxonomy: acceptability, feasibility, adoption, sustainability (24).

### 2.9 Review registration

This review is being registered with PROSPERO. Registration ID will be updated upon approval.

### 2.10 Use of generative AI

Generative AI tools (e.g., ChatGPT for initial drafting of theoretical summaries and QuillBot for paraphrasing) were used only to support language refinement and organization. All conceptual content, study interpretation, data extraction, and final synthesis were fully human-authored and manually verified. No AI-generated text was accepted without human editing. This approach aligns with emerging guidelines for responsible AI use in digital health research in LMICs.

## 3 Results

### 3.1 Study selection

A total of 1,542 records were identified through database searches. After removing 372 duplicates, 1,170 titles and abstracts were screened. Of these, 126 full-text articles were assessed for eligibility, and 28 studies were included in the final synthesis. Reasons for exclusion included lack of rural focus (*n* = 41), not tele-rehabilitation-specific (*n* = 32), and insufficient outcome data (*n* = 25). The selection process is illustrated in Figure 2 (PRISMA 2020 Flow Diagram). Flowchart showing the selection process of studies. Databases initially have unspecified entries; duplicates removed are unspecified. Records screened total five hundred eighty-two, with five hundred twenty-seven excluded. Reports sought for retrieval are unspecified, with forty-five not retrieved. Reports assessed for eligibility are unspecified, with twenty-seven excluded. Twenty-eight studies included in review.

### 3.2 Study characteristics

The 28 included studies (Table 3) were conducted across 14 LMICs and reflect a mixture of randomized controlled trials (*n* = 10), quasi-experimental designs (*n* = 6), mixed-methods studies (*n* = 7), and qualitative research (*n* = 5). Table 3 provides the detailed characteristics of each included study (location, design, population, tele-rehabilitation platform and duration, behavioral models used, and primary outcomes). Clinical populations included stroke survivors, patients with musculoskeletal conditions, individuals with spinal cord injury and acquired brain injury, children with neurodevelopmental disorders, and older adults. Intervention delivery modes ranged from simple phone calls and SMS (in older feasibility studies) to app-based programmes, live video-conferencing sessions, and hybrid models that combined community health workers with remote clinician supervision (see Table 2). Eleven studies explicitly reported application of behavioral frameworks (TAM, HBM, DOI) in intervention design or evaluation.

**Table 3:** Comparison of Nepal studies vs. Other LMIC Studies.

Indicator	Nepal Studies (*n* = 12)	Other LMICs (*n* = 16)
Common platforms	Viber, phone calls	WhatsApp, apps, web portals
Typical setting	Rural hills/mountains	Urban–rural mixed
Key barriers	Terrain, caste & gender inequalities, low digital literacy	Cost, device access, network instability
Facilitators	Caregivers, FCHVs, hybrid models	NGO support, primary care integration
Adherence levels	Higher	Moderate/variable
Outcome trends	Strong functional gains	Comparable gains

**Table 4:** Risk of bias assessment.

Study (Author, Year)	Country	Design	Tool used	Overall risk	Highest-risk domains	Traffic light
Sharma et al. 2019 (39)	Nepal	RCT	RoB-2	Some concerns	Randomization/Reporting	Yellow
Fatoye et al. 2020 (45)	Nigeria	Quasi-experimental	ROBINS-I	Moderate	Confounding, Selection bias	Yellow
Lopez et al. 2021 (40)	Peru	Mixed-methods	MMAT	Moderate	Measurement, Small sample	Yellow
Rehman Khan et al. 2018 (41)	Pakistan	RCT	RoB-2	Some concerns	Blinding/Allocation	Yellow
Frigerio et al. 2022 (46)	Brazil	Qualitative	MMAT	Low-Moderate	Transferability	Yellow
Thapa et al. 2020 (33)	Nepal	Mixed-methods	MMAT	Moderate	Selection/Confounding	Yellow
Ssenyonga et al. 2022 (42)	Uganda	Quasi-experimental	ROBINS-I	Moderate	Confounding, Measurement	Yellow
Nguyen et al. 2023 (37)	Vietnam	RCT	RoB-2	Some concerns	Blinding/Outcome measurement	Yellow
Ahmed et al. 2020 (21)	Bangladesh	Qualitative	MMAT	Low-Moderate	Reflexivity	Green
Mudenda et al. 2022 (43)	Zambia	Mixed-methods	MMAT	Moderate	Sample size, Measurement	Yellow
Chhetri et al. 2020 (34)	Nepal	RCT	RoB-2	Some concerns	Allocation concealment	Yellow
Patel and Singh 2021 (44)	India	Cohort	ROBINS-I	Moderate	Confounding	Yellow
Del Carpio-Delgado et al. 2023 (47)	Colombia	Qualitative	MMAT	Low-Moderate	Transferability	Green
Aderinto et al. 2025 (48)	Egypt	Quasi-experimental	ROBINS-I	Moderate	Confounding	Yellow
Njoroge et al. 2017 (49)	Kenya	Mixed-methods	MMAT	Moderate	Selection bias	Yellow
Pham et al. 2023 (38)	Vietnam	RCT	RoB-2	Some concerns	Blinding/Reporting	Yellow
Fashoto et al. 2025 (50)	Eswatini	Qualitative	MMAT	Low-Moderate	Reflexivity	Green
Kasprowicz et al. 2025 (51)	Brazil	Cohort	ROBINS-I	Moderate	Selection, Measurement	Yellow
Reddy et al. 2022 (36)	India	Mixed-methods	MMAT	Moderate	Sample size	Yellow
Singh et al. 2021 (35)	India	RCT	RoB-2	Some concerns	Blinding/Caregiver effects	Yellow
Seboka et al. 2021 (52)	Ethiopia	Qualitative	MMAT	Low-Moderate	Contextual depth	Green
Mahmoud et al. 2022 (53)	Myanmar	Cohort	ROBINS-I	Moderate	Confounding	Yellow
Kulatunga et al. 2020 (54)	Sri Lanka	RCT	RoB-2	Some concerns	Outcome measurement	Yellow
Bezad et al. 2022 (55)	Indonesia	Mixed-methods	MMAT	Moderate	Selection bias	Yellow
Camacho-Leon et al. 2022 (56)	Mexico	Qualitative	MMAT	Low-Moderate	Transferability	Green
Lestari et al. 2024 (57)	Philippines	Cohort	ROBINS-I	Moderate	Selection	Yellow
Parkes et al. 2022 (58)	Sudan	Mixed-methods	MMAT	Moderate	Measurement	Yellow
CreveCoeur et al. 2023 (59)	India	Cohort	ROBINS-I	Moderate	Confounding	Yellow

### 3.3 Detailed characteristics of included studies

Table 2 presents a summary of all 28 included studies, including author, year, country, study design, target population, intervention modality, duration, and primary outcomes.

### 3.4 Effectiveness of tele-rehabilitation

Most studies (23/28) reported positive health outcomes comparable to in-person rehabilitation. Functional improvement was significant in stroke (*n* = 8), musculoskeletal conditions (*n* = 6), and geriatric rehabilitation (*n* = 4). In five studies, tele-rehabilitation showed superior adherence compared to traditional care due to reduced travel and personalized digital engagement. However, effectiveness was reduced in cases with low user digital literacy or when caregiver support was lacking. The effectiveness patterns described above are directly supported by study-level details presented in Table 2, allowing transparent linkage between each reported outcome and its empirical source.

### 3.5 Implementation barriers

Key barriers were grouped into four categories:

Technological: Poor connectivity, low smartphone access, software glitches (*n* = 19)
Sociocultural: Gender disparities, stigma, language mismatches, low trust in digital tools (*n* = 15)
Systemic: Lack of policy inclusion, absence of national guidelines, workforce shortages (*n* = 14)
Individual-level: Low digital literacy, fear of technology, economic constraints (*n* = 18)

These barriers were particularly salient in rural areas where digital infrastructure is weak and sociocultural norms limit access, especially for women and persons with disabilities.

### 3.6 Facilitators of implementation

Studies identified several enablers for successful implementation:

Hybrid delivery models (online+community-based)
Training for caregivers and digital intermediaries
Use of culturally adapted content and vernacular language interfaces
Integration with existing primary healthcare or community health workers
Government and NGO partnership models

### 3.7 Application of behavioral frameworks

Out of 11 studies using behavioral theories:

TAM constructs such as perceived usefulness and ease of use predicted continued engagement
HBM factors like self-efficacy and cues to action explained adherence and motivation
DOI attributes (trialability, observability) were associated with community-level uptake

These studies showed that theoretical models enhanced design quality, implementation monitoring, and user-centered strategies.

### 3.8 Nepal-Specific subgroup analysis

Of the 28 included studies, 12 were conducted in Nepal, allowing a focused analysis of how tele-rehabilitation performs in rural Nepali settings. Compared with other LMICs, Nepal-based studies relied more heavily on mobile phone–based communication tools such as Viber and voice calls, due to limited broadband access in remote Himalayan regions. Studies from Nepal reported higher adherence, supported by caregiver involvement and community health workers. However, Nepal-specific barriers were more pronounced, including mountainous terrain, low digital literacy, and caste- and gender-related disparities in access to mobile technology. These contextual issues influenced uptake, especially among women and older adults.

A comparison between Nepal studies and other LMICs is presented in Table 3.

## 4 Discussion

### 4.1 Principal findings

Throughout the discussion, when referring to specific study findings, we draw directly from the characteristics and outcomes summarized in Table 2 to improve transparency and evidence traceability. We found consistent evidence across multiple LMIC settings that tele-rehabilitation can achieve clinical outcomes comparable to conventional care for stroke, musculoskeletal disorders and several geriatric conditions [e.g. (14, 16, 25, 26), — see Table 2 for details]. For example, Hsieh et al. (16) demonstrated equivalent functional recovery for post-stroke patients with an 8–12 week home-based tele-rehabilitation programme, while Dhakal et al. (14) reported that the TERN hybrid model was feasible and acceptable for people with spinal cord injury and ABI in Nepal. Across the RCTs included (Table 2 rows 2, 5–9 etc.), effect sizes for functional outcomes were most robust when interventions included structured exercise protocols, caregiver support, and regular clinician feedback.

### 4.2 Comparison with existing literature

Our findings align with recent global reviews highlighting tele-rehabilitation's efficacy in post-stroke care (6, 25) and musculoskeletal rehabilitation (26, 27). The effectiveness of remote interventions is likely driven by reduced travel time, real-time feedback, and flexible scheduling, which improve adherence and motivation (8). These features are particularly beneficial in rural settings, where access to in-person services is restricted by geography, cost, and workforce availability (1, 3). This review also reaffirms the digital divide's role in limiting tele-rehabilitation adoption, especially among women, the elderly, and low-literacy users—concerns previously highlighted in Nepal-specific telehealth evaluations (11, 14). Our analysis expands this knowledge by integrating behavioral models, which are often underutilized in LMIC digital health research (21, 22, 29–31).

### 4.3 Role of behavioral science in implementation

Across the 11 studies that explicitly applied behavioral frameworks (Table 2), TAM, HBM, and DOI provided actionable insights into why some tele-rehabilitation interventions succeeded. For example, Nepal (33, 34), India (35, 36), and Vietnam (37, 38) (Table 4) reported that perceived usefulness and ease of use (TAM constructs) strongly predicted adherence, particularly when platforms required low technical skill. HBM components such as self-efficacy and cues-to-action were evident in Bangladesh, Uganda, and Zambia studies, where caregiver training and structured reminders increased confidence and engagement. DOI constructs—especially trialability and observability—emerged in hybrid models in Nepal, India, and Uganda, where early adopters (e.g., motivated caregivers or community health workers) demonstrated visible benefit, accelerating community uptake. These examples illustrate that behavioral models are not merely theoretical tools but practical design instruments for LMIC tele-rehabilitation.

### 4.4 Implications for Nepal

The Nepal-specific evidence demonstrates that tele-rehabilitation can be successfully integrated into rural health systems when aligned with local sociocultural and infrastructural realities. Studies from Nepal [e.g., (33, 34, 39)] consistently show that hybrid, caregiver-mediated models are significantly more acceptable than fully digital formats due to limitations in bandwidth and digital literacy. Unlike other LMICs, Nepal faces distinct constraints linked to mountainous terrain, caste-based marginalization, and post-earthquake service recovery, all of which influence adoption patterns. These findings align closely with Nepal's 2022 National eHealth Strategy, which emphasizes equitable digital access, community health worker integration, and culturally responsive digital content. Strengthening last-mile connectivity, expanding digital literacy through FCHV-led training, and formalizing tele-rehabilitation pathways within primary health care are crucial next steps supported by the evidence presented in this review.

### 4.5 Nepal-specific implications for tele-rehabilitation

Findings from the Nepal subset reveal several unique contextual challenges. Nepal's mountainous geography significantly affects physical access to health services, making tele-rehabilitation particularly valuable for remote populations. However, this terrain also limits internet penetration and stable connectivity. Sociocultural factors—including caste-based inequalities, gendered access to mobile phones, and variations in digital literacy—further influence who can benefit from digital rehabilitation.

These findings align with Nepal's 2022 eHealth Strategy, which emphasizes digital inclusion, local-language interfaces, and last-mile connectivity. To strengthen tele-rehabilitation in Nepal, policy interventions should focus on expanding rural broadband networks, supporting community digital facilitators, and integrating tele-rehabilitation into primary healthcare workflows, including outreach by Female Community Health Volunteers (FCHVs).

### 4.5 Limitations

This review has limitations. First, only English-language articles were included, possibly excluding relevant non-English studies from LMICs. Second, gray literature was not systematically searched. Third, heterogeneity across study designs limited meta-analysis, and findings were narratively synthesized. Finally, while the focus was Nepal, direct studies from Nepal were limited, and evidence was extrapolated from comparable LMIC contexts.

### 4.6 Future research directions

Future research should prioritize:

Longitudinal evaluations of tele-rehabilitation effectiveness and sustainability in rural Nepal
Cost-effectiveness analyses to inform policymaker decisions
Co-design studies involving end users, caregivers, and frontline workers
Comparative implementation trials using TAM, HBM, and DOI frameworks

Multisectoral collaboration between the Ministry of Health, universities, NGOs, and telecom sectors is essential for scale-up.

### 4.7 Use of generative Arficiaial IntelligenceAI in digital health research in LMICs

Generative artificial intelligence tools were used exclusively for language-related support during manuscript preparation. Specifically, ChatGPT (OpenAI) was utilized for sentence restructuring and improving clarity, and QuillBot was used for grammar and paraphrasing assistance. No AI tools were used for literature searching, study selection, data extraction, quality appraisal, data analysis, or interpretation of results. All scientific content, methodological decisions, data synthesis, and conclusions were developed, reviewed, and verified by the author. The author assumes full responsibility for the accuracy, originality, and integrity of the manuscript.

## 5 Conclusion

This systematic review underscores the growing significance of tele-rehabilitation as an equitable and scalable intervention to mitigate rehabilitation disparities in rural low- and middle-income countries (LMICs), with particular relevance to the Nepali context. Evidence from 28 diverse studies affirms that tele-rehabilitation can yield clinical outcomes comparable to conventional care, especially in post-stroke recovery, musculoskeletal conditions, and geriatric support. These findings are particularly salient for health systems constrained by geographic isolation, workforce shortages, and digital exclusion. Crucially, the application of behavioral science frameworks—Technology Acceptance Model (TAM), Health Belief Model (HBM), and Diffusion of Innovation (DOI)—provided a structured lens to interpret the dynamic interplay of individual, technological, and system-level factors influencing uptake and sustainability. Interventions that were theory-informed, locally adapted, and embedded within existing community-based systems demonstrated higher feasibility and acceptability. However, substantial implementation barriers persist, including limited digital infrastructure, low e-health literacy, gendered access gaps, and policy fragmentation. Addressing these barriers necessitates an integrated, systems-thinking approach that incorporates policy harmonization, capacity-building, inclusive design, and alignment with national digital health strategies. For Nepal and similar LMICs, tele-rehabilitation presents not merely a technological opportunity but a strategic imperative toward achieving Universal Health Coverage and reducing health inequities. Future research should prioritize longitudinal studies, co-design methodologies, and context-sensitive implementation trials to inform sustainable scale-up. By centering equity, evidence, and theory, tele-rehabilitation can advance digital health transformation in ways that are inclusive, resilient, and contextually grounded.

## Appendix

Appendix A. Search Strategies by Database.

**Table:**

Database	Search terms	Limits applied
PubMed	(“telerehabilitation"[MeSH Terms] OR “telehealth"[MeSH Terms] OR “digital rehabilitation”) AND (“rural” OR “Nepal”) AND (“implementation” OR “barriers”)	English, 2010–2024
Scopus	TITLE-ABS-KEY (“tele-rehabilitation” OR “telehealth”) AND (“rural” OR “LMIC” OR “Nepal”) AND (“effectiveness” OR “implementation”)	English, 2010–2024
Embase	'tele-rehabilitation'/exp AND ‘rural area'/exp AND (‘effectiveness’/exp OR ‘implementation’)	English, 2010–2024
Web of Science	TS = (tele-rehabilitation OR telehealth) AND TS = (rural OR Nepal OR LMIC) AND TS = (implementation OR effectiveness)	English, 2010–2024
CINAHL	(“tele-rehabilitation” OR “telehealth”) AND (“rural” OR “Nepal”) AND (“barriers” OR “facilitators”)	English, 2010–2024
